# Intermingling of Chromosome Territories in Interphase Suggests Role in Translocations and Transcription-Dependent Associations

**DOI:** 10.1371/journal.pbio.0040138

**Published:** 2006-04-25

**Authors:** Miguel R Branco, Ana Pombo

**Affiliations:** **1**MRC Clinical Sciences Centre, Faculty of Medicine, Imperial College London, Hammersmith Hospital Campus, London, United Kingdom; Adolf Butenandt InstituteGermany

## Abstract

After mitosis, mammalian chromosomes partially decondense to occupy distinct territories in the cell nucleus. Current models propose that territories are separated by an interchromatin domain, rich in soluble nuclear machinery, where only rare interchromosomal interactions can occur via extended chromatin loops. In contrast, recent evidence for chromatin mobility and high frequency of chromosome translocations are consistent with significant levels of chromosome intermingling, with important consequences for genome function and stability. Here we use a novel high-resolution in situ hybridization procedure that preserves chromatin nanostructure to show that chromosome territories intermingle significantly in the nucleus of human cells. The degree of intermingling between specific chromosome pairs in human lymphocytes correlates with the frequency of chromosome translocations in the same cell type, implying that double-strand breaks formed within areas of intermingling are more likely to participate in interchromosomal rearrangements. The presence of transcription factories in regions of intermingling and the effect of transcription impairment on the interactions between chromosomes shows that transcription-dependent interchromosomal associations shape chromosome organization in mammalian cells. These findings suggest that local chromatin conformation and gene transcription influence the extent with which chromosomes interact and affect their overall properties, with direct consequences for cell-type specific genome stability.

## Abstract 2

The authors apply a novel high-resolution in situ hybridization method that preserves chromatin nanostructure and show that chromosome territories intermingle significantly in the nucleus of human cells.

## Introduction

Chromatin organization in the cell nucleus influences gene expression, DNA replication, damage, and repair. When the interphase nucleus forms, chromosomes partially decondense but still occupy distinct territories [ 1], which have nonrandom radial positions that are conserved through evolution [ 2– 5]. Current models suggest that chromosome territories (CTs) are separated by an interchromatin domain (ICD), rich in the nuclear machinery for nucleic acid metabolism. According to the ICD model, active genes are found in direct contact with the ICD [ 6], and occasionally fine chromosome fibers extend into this domain, where rare interchromosomal interactions may occur [ 1, 7– 9]. However, a physical separation between CTs is not supported by data on translocation frequencies and chromatin dynamics. Simulations of chromosome translocations based on models of chromosome organization have suggested the existence of a significant degree of intermingling between CTs [ 10– 12]. Furthermore, in vivo studies have shown that although chromatin domains are relatively stable [ 13], individual loci show diffusion dynamics constrained to approximately 0.4 μm [ 14– 16] and can exhibit movements as large as 1.5 μm [ 15]. This argues against a strict localization of chromatin within a CT that would prevent extensive intermingling.

Recently, specific associations between loci on different chromosomes have been reported [ 17, 18], which are reminiscent of intrachromosomal clustering that is essential for correct gene expression [ 17, 19– 23]. It remains unclear whether these are just a few rare examples of interchromosomal associations that occur via chromatin fibers that extend from their own CTs or whether a greater potential exists for interactions through more extensive intermingling of chromosomes in interphase. Such interactions, if abundant, would be expected to determine chromosome organization and thereby influence the range of translocations that occur in each cell type.

Previous data on chromosome morphology and organization have mainly originated from painting of whole chromosomes by fluorescence in situ hybridization (FISH) in three-dimensional (3D) nuclei. However, 3D-FISH is known to provide low spatial resolution and to compromise chromatin organization at the local level [ 24]. We have developed a novel FISH procedure for ultrathin cryosections (approximately 150 nm thick; cryo-FISH) of well-fixed [ 25], sucrose-embedded cells, that maximizes chromosome-painting efficiency, provides high resolution, and simultaneously preserves chromatin nanostructure. We show here that chromosomes intermingle significantly in interphase nuclei of human cells, arguing against the presence of an interchromosomal domain that separates CTs. The extent with which particular pairs of CTs intermingle correlates with the frequency of chromosome translocations in the same cell type [ 26]. Furthermore, we show that blocking of transcription changes the pattern of intermingling while preserving general chromosome properties, such as compaction and radial position, indicating that transcription-dependent associations between CTs are frequent enough to influence chromosome organization. In line with this view, we find that activation of the MHC class II gene cluster by interferon-gamma (IFN-γ) causes an increased colocalization of this locus with other chromosomes, concomitant with the relocation to a more external position in relation to its own CT [ 27].

## Results/Discussion

### Chromosome Territories Intermingle

Previous studies of chromosome organization during interphase have relied on the painting of chromosomes in whole nuclei, in conditions that compromise painting efficiency to preserve three-dimensionality. However, even in the best conditions, the nanostructure of chromatin at the level of single chromatin domains is lost [ 24]. To overcome this limitation, we developed a FISH procedure (cryo-FISH) using ultrathin cryosections of cells fixed under stringent conditions [ 25].

**Figure 1:**
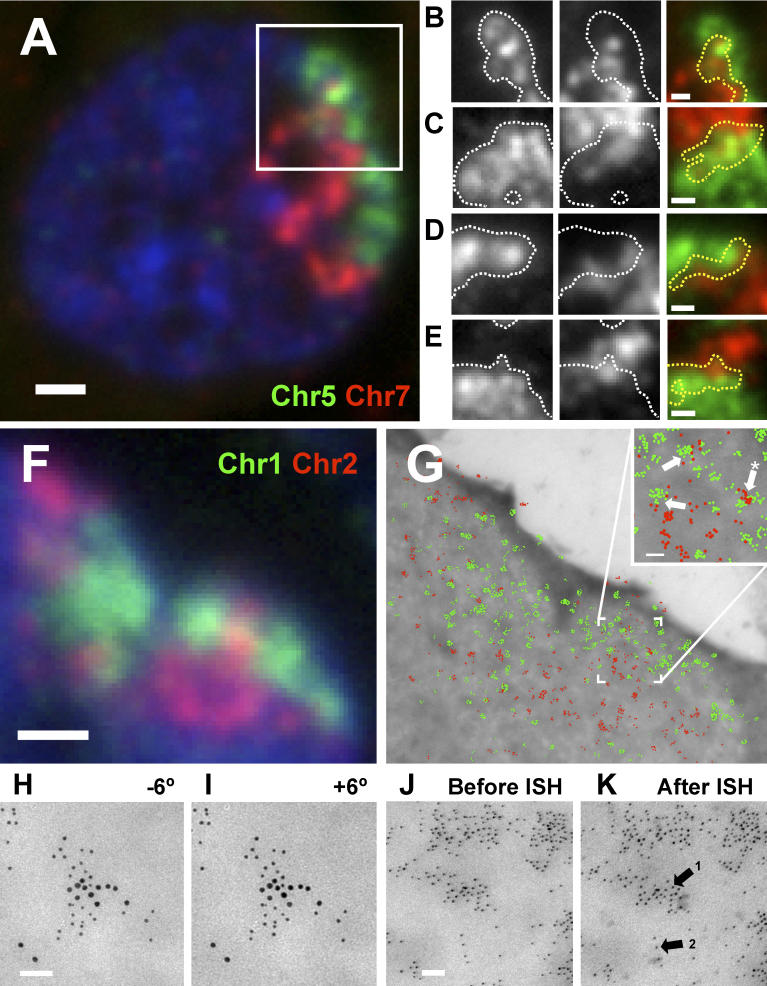
Chromosome Territories Intermingle in the Nucleus of Human Cells Chromosomes were painted in cryosections (approximately 150 nm thick) of human lymphocytes and visualized by fluorescence microscopy (A–F) or indirectly immunolabeled with gold particles before imaging by electron microscopy (G–I). (A) Example of a nuclear section showing intermingling between Chromosomes 5 and 7. (B–E) Intermingling is best seen in gray-scale images after the mask for one chromosome (white line) is overlaid on the image of the other chromosome. The intersection between masks for both chromosomes is shown in the merged images (yellow line), representing areas of intermingling. Fluorescence intensity line scans of relevant areas in images (B–E) can be in Figure S1. (F and G) Five- and 10-nm gold particles labeling Chromosomes 1 and 2 (G; pseudo-colored green and red, respectively) are intermingled within the intersections identified on the light microscope in the same nuclear section (F). Gold particles labeling different CTs can be found in close proximity within areas of intermingling (G, arrows in inset). (H and I) Stereoviews of a region of colocalization (*) between Chromosome 1 (5 nm gold) and 2 (10 nm gold) were obtained by collecting images tilted −6° (H) and +6° (I) relative to the *z*-axis. 3D visualization shows that differently-sized particles lie adjacent in the same *z* planes. (J and K) Histone H2B was indirectly immunolabeled with 5-nm gold particles and imaged on the EM before (J) and after (K) mock FISH; gold particles are spatially preserved in both heterochromatic (arrow 1) and euchromatic (arrow 2) regions. Bars: (A and F) 1 μm; (B–E) 0.5 μm; (G–K) 50 nm.

To test for chromosome intermingling we cohybridized pairs of whole chromosome paints to sections of phytohemagglutinin-activated human lymphocytes ( Figure 1). Binary masks were obtained for each CT and their intersections used to identify areas of colocalization ( Figure 1B– 1E). Fluorescence intensity profiles confirm that these areas contain DNA from two chromosomes ( Figure S1). Intermingling was detected for all chromosome pairs analyzed in these primary cells, but also in other human cell types (resting lymphocytes, HeLa cells, and primary fibroblasts; unpublished data).

Due to the low resolution of the light microscope (LM; at best 200 nm in the *x* and *y* axes), we tested by electron microscopy (EM) whether DNA from different chromosomes is found in close proximity within areas of intermingling ( Figure 1G). After FISH, sections were first imaged on the LM to locate areas of intermingling ( Figure 1F), before indirectly immunolabeling the fluorochromes in the paints (FITC and rhodamine) with 5- and 10-nm gold particles, respectively ( Figure 1G). CTs labeled by immunogold particles strongly correlate with the corresponding LM image. Areas of intermingling identified by LM were found to contain colocalized gold particles labeling different chromosomes ( Figure 1G, inset, arrows; more than ten sections with intermingled CTs analyzed), showing that they are sufficiently close to interact at the molecular level. Stereoviews of regions of intermingling show that gold particles of different sizes are found at the same z-planes ( Figure 1H and 1I; eight regions of intermingling in four nuclear profiles analyzed), such that the intersection between CTs cannot be simply explained by distant territories that overlap within the thickness (approximately 150 nm) of the section.

We next tested whether intermingling could result from artefactual chromatin disruption due to the harsh cryo-ISH procedure, in spite of the stringent fixation used. We compared the distribution of histone H2B, DNA, and sites of transcription labeled with Br-UTP, before and after ISH, and found that intermingling or the close proximity of gold particles labeling different chromosomes could not be explained by loss of fine chromatin structure during the procedure ( Figures 1J, 1K, and S2). Strikingly, the position of gold particles labeling histone H2B remains constant before and after FISH ( Figure 1J and 1K).

To determine whether cryo-ISH had simply revealed the rare interactions between looped chromatin or showed more extensive intermingling of CTs, we measured how much of one CT intermingles with all others. We labeled sections with a Chromosome 3 paint, together with a probe that hybridizes with all other chromosomes ( Figure S3), and found that 41% of the volume of Chromosome 3 intermingles with the remaining genome. Although this argues against the existence of an interchromatin space that separates CTs, it remained possible that intermingling involved only loops of less condensed chromatin [ 1, 7, 8]. Therefore, we asked whether chromatin concentration within areas of intermingling is lower to that within a CT. We compared the fluorescence intensity of general DNA dyes (DAPI or TOTO-3 after RNase treatment) in intermingled regions of a CT with nonintermingled regions, or with the whole nucleoplasm, and found no significant differences (ratios of 1.12 ± 0.20 and 1.02 ± 0.52, respectively; *n* = 32). This shows that similar average DNA concentrations are present in intermingled and nonintermingled regions, indicating that chromatin has similar average properties in both areas. In fact, we also observed mixing of chromatin fibers within a CT (“intramingling”) between both arms of a chromosome (approximately 10% of Chromosome 3 volume; see also [ 28]). Therefore, regions of higher accessibility to transcription and pre-mRNA processing factors do not preferentially locate between CTs but are more uniformly distributed throughout the nucleoplasm, as shown previously [ 29, 30].

### Different Extents of Chromosome Intermingling between CTs Correlate with Translocation Frequencies

**Figure 2:**
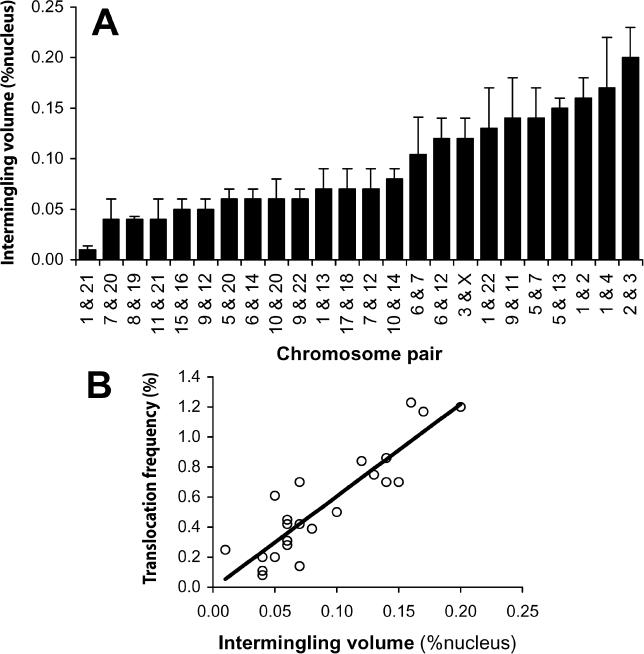
CT Intermingling Correlates with Translocation Potential Intermingling volumes were measured for 24 pairs of chromosomes in human lymphocytes (A) and plotted against the respective translocation frequency in the same cell type ( Table S1) [ 26] (B), showing a highly significant correlation ( *p* < 0.0001). Error bars represent standard deviations.

Chromosome intermingling has been suggested by modeling of translocation frequencies [ 10– 12], but not previously visualized, except for rare interactions [ 9]. A prediction of such models is that the extent of intermingling between each pair of chromosomes should be reflected in their translocation potential. We therefore measured the intermingling volumes for 24 pairs of chromosomes in activated human lymphocytes using a simple stereological principle (see Materials and Methods). Chromosome pairs were selected to reflect a wide range of translocation frequencies as measured in the same cell type by Arsuaga et al. [ 26] ( Table S1). The fraction of one chromosome (both homologs) that intermingles with any of the other 22 chromosomes is, on average, 2.1 ± 1.1%. This would correspond to 46% of each chromosome being intermingled with the rest of the genome (2.1% × 22 chromosomes), which is in agreement with the experimental value of 41% obtained for Chromosome 3. To obtain absolute values that are independent of CT volume, we expressed intermingling as a percentage of the nuclear volume ( Figure 2A). These values are representative of the average across the cell population, thus taking into account the frequency of CT association and the extent of intermingling when they are associated. Intermingling volumes between individual chromosome pairs vary by 20-fold ( Figure 2A), and show statistically significant differences ( *p* < 0.0001, ANOVA).

A previous study has shown that two cell-type specific translocations are associated with closer proximity between the CTs involved [ 31]. We therefore asked whether the extent of intermingling, rather than CT association alone, is predictive of chromosome stability on a larger scale. Intermingling volumes determined above were plotted against the frequency of chromosome translocations measured in the same cell type after ex vivo exposure to ionizing radiation [ 26] ( Table S1). We found a highly significant correlation between the extent of intermingling and translocation frequency ( *p* < 0.0001; Figure 2B), such that higher levels of CT intermingling increase the chances of double-strand breaks (DSBs) being involved in rearrangements with other chromosomes. This result suggests that DSBs formed within areas of intermingling are more likely to translocate, although it does not exclude movement and clustering of DSBs formed elsewhere as a possible pathway for translocation genesis [ 32].

Given the proportionality between intermingling and translocation frequency, and the fact that the existing data for translocation frequencies covers the whole genome [ 26] ( Table S1), it is possible to estimate the total CT intermingling in the cell nucleus (i.e., for all chromosomes; see Protocol S1). Using either the intermingling volumes obtained for pairs of chromosomes or between Chromosome 3 and the remaining chromosomes, it can be estimated that intermingled regions account for 19% of the nuclear volume. As areas of intermingling contain sequences from at least two chromosomes, this value is strikingly consistent with an average of approximately 40% of the volume of each chromosome being intermingled with the remaining genome. Although the existence of triple intermingling (not accounted for in the estimation) would decrease this value, measurements involving three chromosomes (Chromosomes 1, 2, and 3) showed that it occurs only to a relatively small extent (0.01% of the nucleus, *n* = 90 nuclear profiles). On the other hand, intermingling between homologues (also not accounted for) would increase the total chromosome intermingling. The contribution of repetitive sequences (unlabeled by chromosome painting) such as those present in centromeres will depend on whether these sequences are excluded from intermingling regions.

### Chromosome Intermingling Is Influenced by Transcription-Dependent Interactions between Chromosomes

**Figure 3:**
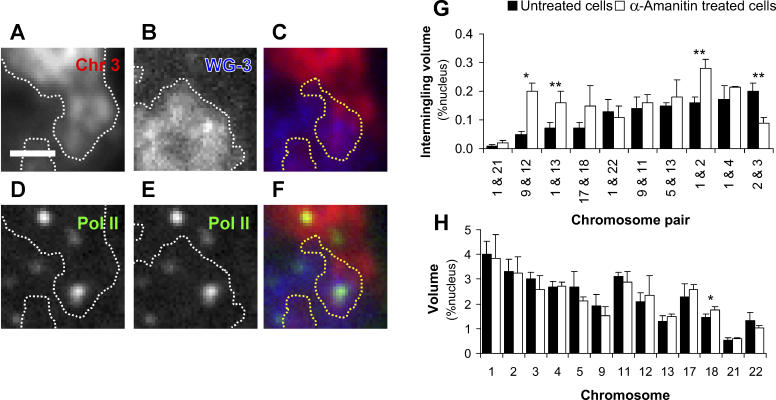
Chromosome Intermingling Is Influenced by Transcription-Dependent Interactions (A–F) Immuno-FISH labeling shows the active form of PolII in regions of intermingling. Serine2-phosphorylated PolII was immunolabeled with H5 antibodies and AlexaFluor488, before hybridization with paints for Chromosome 3 and all other chromosomes (WG-3). A magnified view of Chromosome 3 (A) and the remaining chromatin (B) is shown, which contains a region of intermingling (C). Active PolII is found within masks delineating Chromosome 3 territory (D), the remaining chromatin (E) and regions of intermingling (F). PolII sites are not excluded or enriched in areas of intermingling (see Figure S4). Bar: 1 μm. (G) Measurements of intermingling for ten chromosome pairs in control and α-amanitin–treated lymphocytes reveals changes in intermingling volumes for four of ten pairs analyzed, showing that the extent of intermingling is affected by ongoing transcription. (H) Differences in intermingling are not due to altered CT volumes, as these did not change after treatment with α-amanitin for 12 of 13 chromosomes analyzed. Error bars represent standard deviations (* *p* < 0.05, ** *p* < 0.01).

The extent of CT intermingling may result solely from passive mixing of chromatin fibers or may also be influenced by specific interactions at the molecular level, which can result from local tethering of distant chromatin fibers. Chromosome position in the cell nucleus depends on cell type [ 31] and on global gene activity [ 5], suggesting that the transcription status of particular sets of genes may directly influence chromosome organization. Expression-dependent, long-range DNA interactions in *cis* between loci up to several Mbp apart [ 17, 19– 23] and between different chromosomes [ 17, 18, 33, 34] are likely to be involved, but little is known of the mechanism that bring these interactions about and whether they can influence chromosome organization. One likely candidate to mediate or stabilize such interactions is ongoing transcription, via RNA polymerase (Pol) clusters (known as transcription factories) [ 17, 35, 36], to which transcription units and probably regulatory regions can be tethered. If DNA-tethering via transcription factories contributes to CT interactions, the latter should be detected in areas of intermingling. Indeed, sites containing the serine2-phosphorylated (active [ 37]) form of PolII are present within areas of intermingling between Chromosome 3 and all other chromosomes ( Figure 3A– 3F). Quantitative analyses show that PolII sites are neither diminished nor enriched within regions of intermingling in relation to the average site density in the whole nucleus ( Figure S4). This analysis also confirms that active transcription factories are present within chromosome territories as shown previously [ 29, 30].

To analyze the effect of ongoing transcription and polymerase clustering on the pattern of intermingling between specific pairs of chromosomes, we treated human activated lymphocytes with α-amanitin in conditions that inhibit PolII-driven transcription within 1 h of treatment, significantly reduce the level of serine2-phosphorylated PolII (unpublished data; [ 38]), and disassemble transcription factories (P. V. Guillot, S. Martin, F. Antunes, D. L. Bentley, A. Pombo, unpublished data; see also [ 39]). We then measured the intermingling and CT volumes for ten pairs of chromosomes that exhibit a wide range of intermingling volumes in untreated cells. We found that transcription inhibition significantly decreased intermingling for one pair and increased it for three of the ten pairs ( Figure 3G). Such changes indicate that there are direct transcription-dependent interactions between CTs that are strong enough to influence chromosome conformation. To rule out indirect effects of transcription inhibition from changes in the volume of the nucleus or CTs, we measured these parameters before and after α-amanitin treatment and found that both nuclear (not shown) and CT ( Figure 3H) volumes did not change, except for Chromosome 18, which had only a small but statistically significant increase (from 1.4% to 1.7% of nuclear volume, *p* = 0.047). The radial position of these CTs also remained constant (M. Branco, T. Branco, and A. Pombo, unpublished data). The absence of indirect effects is further validated by the fact that the total intermingling of Chromosome 3 with all other CTs remained the same after α-amanitin treatment (40% of Chromosome 3; *n* = 88 nuclear profiles), even though the intermingling of Chromosome 3 with Chromosome 2 decreased. Taken together these results show that transcription-dependent chromatin interactions influence chromosome arrangements, determining specific intermingling “partners” according to the cell's repertoire of active genes. The fact that the total extent of CT intermingling for Chromosome 3 remains unaltered upon transcription inhibition further supports the conclusion that CT intermingling is not simply due to rare interchromosomal interactions at the surface of territories but rather a property of chromatin, as recently proposed [ 14, 40].

### Chromosome Decondensation Allows for Increased Intermingling

**Figure 4:**
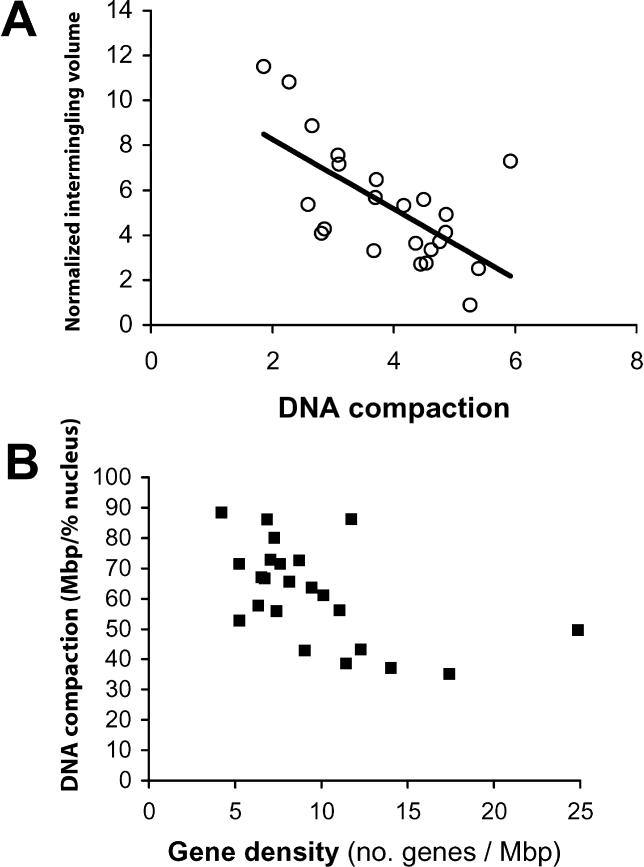
Less Compact Chromosomes Are More Gene-Rich and Tend to Intermingle More The normalized intermingling volumes for each pair of CTs were plotted against the product of their DNA compaction ratios (A), which were obtained by calculating the respective DNA content per unit of CT volume ( Figure S5). Individual DNA compaction ratios were plotted against the respective gene density (B). The negative correlations observed in both cases reveal that gene-rich chromosomes tend to be less compact, creating a higher potential for nonspecific intermingling. Error bars represent standard deviations.

Passive mixing of chromatin fibers from different chromosomes is also likely to contribute to intermingling. To analyze the effect of passive mixing, we measured the volumes for all CTs in human lymphocytes and found that although volume is generally proportional to DNA content, several values deviate significantly from the regression curve, resulting in different compaction ratios for each chromosome ( Figure S5). Therefore, we compared CT intermingling with DNA compaction (defined here as number of base pairs per unit volume of CT) and found that less-condensed CTs tend to exhibit a higher proportion of intermingling ( Figure 4A). We also find that DNA compaction negatively correlates with gene density ( Figure 4B), as previously suggested [ 41], such that gene-rich CTs are more decondensed than gene-poor ones. Calculation of number of genes per unit volume of CT shows that genes are similarly spaced in the nucleus, irrespective of the gene density in the linear sequence of a chromosome (unpublished data); this is consistent with the uniform distribution of transcription sites throughout the nucleoplasm of HeLa cells [ 36] ( Figure S2).

### Active Genes Can Be Found within Neighboring CTs

**Figure 5:**
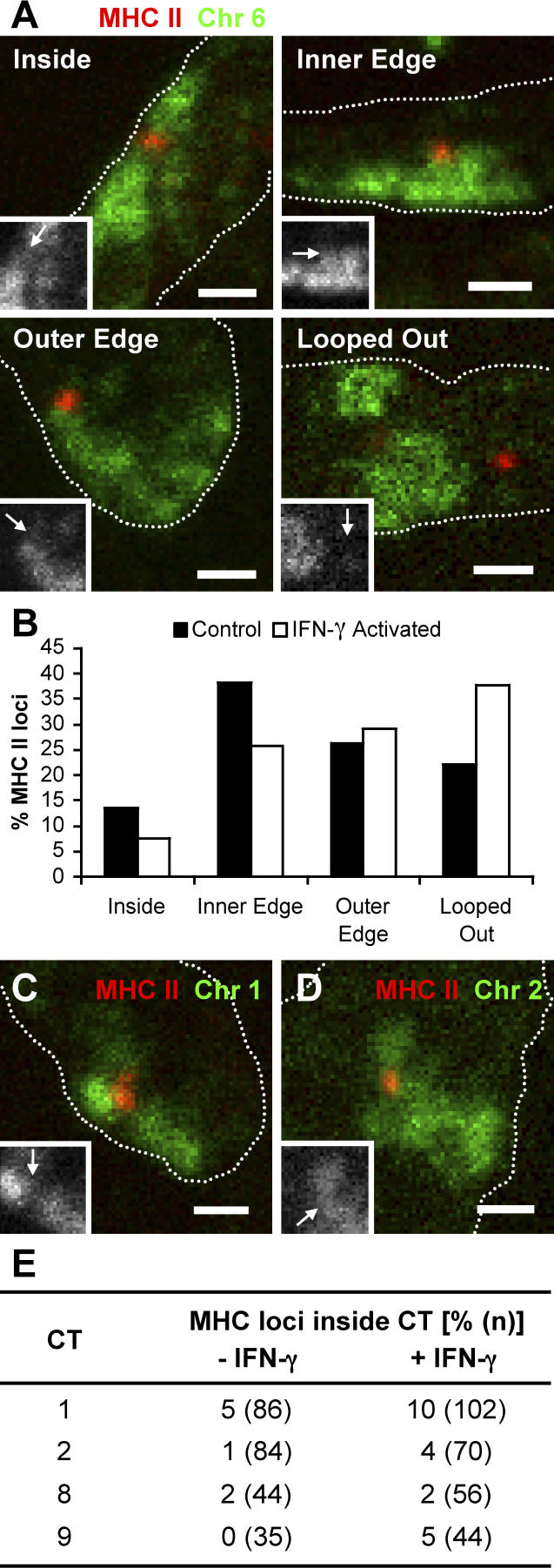
The MHC II Locus Is Found within Other CTs upon IFN-γ Activation (A and B) A BAC probe for the MHC II locus (red) was cohybridized with a Chromosome 6 paint (green) in cryosections of control and IFN-γ activated MRC5 human lung fibroblasts (nuclear edge outlined by dotted line). Insets show the position of the MRC II locus (arrows) in relation to its CT (in gray scale) (A). The positions of the MRC II loci were scored into four different categories. Loci found “inside” or “looped out” were easily classified; loci near the edge of the CT were divided into “inner edge” and “outer edge,” depending on whether they appeared more internal or external in relation to the remainder of the CT. Upon IFN-γ activation, the MHC II locus relocates to a more external position in relation to its CT, when compared with control cells (B, *p* = 0.02, two-tailed χ ^2^ test, *n* = 118 and 117 loci for control and IFN-γ activated cells, respectively), as described before [ 27]. Note that due to the flatness of fibroblast cells, nuclear profiles from random sections are often elongated. (C–E) The MHC II BAC probe (red) was cohybridized with paints for Chromosomes 1 (C, green), 2 (D, green), 8, or 9 (not shown) and the number of MHC II loci found within each of these CTs was scored in both control and IFN-γ activated cells (E). Upon activation, the MHC II locus is more likely to be found within one of Chromosomes 1, 2, or 9, when compared with control cells ( *p* = 0.038, two-tailed Fisher's exact test using pooled data from the three chromosomes), whereas no difference in association is detected if Chromosome 8 is included in the analysis ( *p* = 0.052, two-tailed Fisher's exact test using pooled data from all four chromosomes).

The location of active genes relative to their CTs has been a main focus in the study of chromosome organization. Early reports looking at a small number of genes suggested that they preferentially locate at the chromosome periphery [ 42, 43], whereas analysis of nuclear transcription after labeling of nascent transcripts with Br-UTP showed incorporation throughout the chromosome territory (CT) [ 29, 30] (see also Figures 3 and S4). Given the change in the intermingling pattern observed after transcription inhibition ( Figure 3G), we asked whether active genes that tend to lie at the periphery of chromosome territories can be found within other territories. For this purpose we chose the MHC class II locus, which is known to become more externally positioned relative to its CT upon activation with IFN-γ, in MRC5 human lung fibroblasts [ 27]. Cryosections from control and IFN-γ activated cells were hybridized with a Chromosome 6 paint and a BAC that lies within the MHC class II gene cluster, and the position of the MHC II signal scored relative to the CT signal ( Figure 5A). We find that the locus is already present away from the main body of Chromosome 6 territory in 22% of untreated cells (versus 13% reported by Volpi et al. [ 27]), probably due to the improved resolution of the cryo-FISH approach ( Figure 5B). As described before, IFN-γ activation promoted repositioning of the MHC II locus toward a more external position in relation to Chromosome 6 [ 27] ( Figure 5A and 5B). It is worthwhile noting that only one in four of the looped-out MHC II loci were painted by the Chromosome 6 probe, despite our improved FISH procedure; this suggests that our estimation of intermingling values is conservative. To test whether the locus can be found within other CTs, we cohybridized the MHC II BAC probe with chromosome paints for four other chromosomes (1, 2, 8, and 9; Figure 5C– 5E). In nonactivated cells, the gene was seldom found within other CTs, as expected from the more internal position relative to its own territory ( Figure 5E). However, after IFN-γ activation, and concomitant with the externalization of the locus from Chromosome 6 territory, we detected an increase in association with three of the four chromosomes analyzed ( Figure 5E). The preferential association with some chromosomes (1, 2, and possibly 9) but not others (Chromosome 8) suggests some specificity that may simply result from preferential CT neighborhoods and/or reflect an association with particular loci in some chromosomes.

In summary, the data show that both physical properties and functional interactions between CTs determine chromosome intermingling. This is further supported by comparison of our experimental values of CT intermingling with those obtained from calculations of chromosome intermingling using theoretical models of chromosome organization that only take into account physical properties (G. Kreth and C. Cremer, unpublished data).

**Figure 6:**
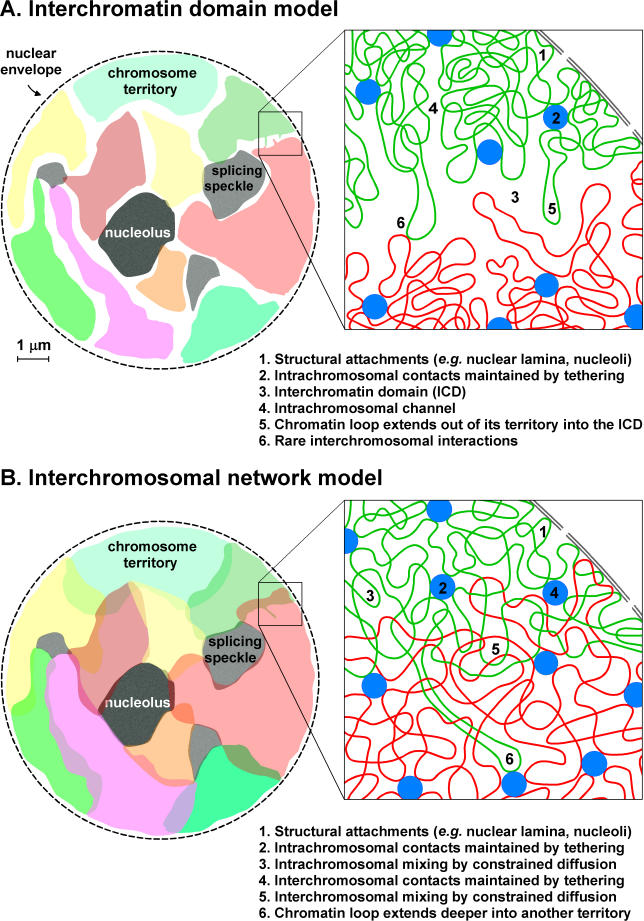
ICD and Interchromosomal Network (ICN) Models of Chromatin Organization in Mammalian Nuclei (A) In the ICD model, chromatin from different chromosomes is separated by an ICD compartment rich in nuclear machinery. Active genes are in direct contact with the ICD compartment, as they lie at the surface of CTs or intrachromosomal channels. Rare chromatin loops extending from CTs may invade the ICD space, which is predicted to contain little or no chromatin. Misrejoining between open ends from DSBs on different chromosomes is less likely as broken ends must travel significant distances. (B) In the ICN model, chromatin from different chromosomes is not separated by a compartment but is allowed to expand into the surrounding territories; the presence of adjacent chromosomes, the nuclear membrane, and larger nuclear compartments restricts the amount of intermingling. DNA sequences along chromosomes will have different properties that determine their compaction, mobility, and affinity to specific nuclear components (1). Despite the different local levels of compaction, the global average properties of chromatin in areas of intermingling will be similar to those found within a CT. Rare chromatin loops extending from a CT can invade neighboring CTs (6). Functional associations that correlate with active and inactive states of transcription (2 and 4), including those involving clustering of active RNA polymerases on transcription factories, determine local arrangements within and between chromosomes, that influence the large-scale organization of chromosomes in each cell type. In the ICN model, DSBs formed in regions of intermingling are more likely to produce interchromosomal rearrangements, whereas DSBs elsewhere in the chromosome are more likely to produce intrachromosomal rearrangements.

Current models of chromosome organization ( Figure 6A) are not consistent with the high frequency of complex chromosomal aberrations [ 10– 12] or with the fact that chromatin is dynamic, showing diffusion-like movements [ 14– 16, 44]. The analysis of chromosome intermingling in human lymphocytes, for which quantitative translocation data across the whole genome are available, has allowed us to reconcile these different models in one unified view ( Figure 6B). Chromosomes occupy territories that intermingle with one another, mostly at their boundaries. These interactions have direct consequences for chromosome stability, as the proximity of DSB in regions of intermingling will inevitably facilitate interchromosomal rearrangements. Ongoing transcription influences the degree of intermingling between specific chromosomes, probably by stabilizing associations between particular loci. Intrachromosomal associations will favor chromosome discreteness whereas interchromosomal ones will favor intermingling. Such interactions are likely to depend on the transcriptional activity of the loci, and therefore be cell type specific, as it has been shown for both intrachromosomal [ 17, 19, 21– 23] and interchromosomal [ 18, 33, 34] associations. Given the fact that a significant fraction of chromatin is found intermingled in the interphase nucleus, many more interchromosomal associations are likely to exist. We propose that such a network of interactions contains epigenetic information that will at the same time result from and influence the specific transcriptome and sets of chromatin modifications present in each cell type and determine the range of potential chromosome rearrangements.

## Materials and Methods

### Cell culture, fixation, and cryosectioning

Human female peripheral blood mononuclear cells were purified by a Leuco-Sep Separation Media (Human; Harlan Sera-Lab, Loughborough, United Kingdom) density gradient centrifugation (500 *g*, 30 min) and grown in RPMI-1640 medium containing 5% heat-inactivated FCS, 5 mM sodium pyruvate, 2 mM glutamine, 50 IU/ml penicillin, 50 μg/ml streptomycin (all from Life Technologies, Paisley, United Kingdom), 50 μg/ml β-mercaptoethanol (Sigma, Dorset, United Kingdom), and 5 μg/ml phytohemagglutinin (Sigma) for 72 h. Cells were incubated ±50 μg/ml α-amanitin (Sigma) for 6.5 h. MRC5 human lung fibroblasts (E CACC) were grown in RPMI-1640 medium containing 10% heat-inactivated FCS and incubated ±500 U/ml recombinant human IFN-γ (Roche Diagnostics, East Sussex, United Kingdom) for 20 h; final confluency was 70% to 80%. IFN-γ activation was confirmed by the increase in the number of PML bodies [ 45] observed after immunolabeling (see below).

For the preparation of cell blocks for cryosectioning, cells were fixed in 4% and then 8% paraformaldehyde in 250 mM HEPES (pH 7.6) (10 min and 2 h, respectively) [ 25]. Cell pellets were embedded in 2.1 M sucrose in PBS and frozen in liquid nitrogen as described previously [ 35]. Cryosections (140 to 180 nm in thickness, deduced from interference color) were cut using an UltraCut UCT 52 ultracryomicrotome (Leica, Milton Keynes, United Kingdom), captured in sucrose drops, and transferred to coverslips (for LM) or nickel grids coated with 0.5% Formvar (for EM). Sections on sucrose drops were stored at −20 °C.

### Cryo-FISH

Directly labeled (rhodamine, Texas Red, or FITC) whole human chromosome paints (Qbiogene, Cambridge, United Kingdom), or a biotin-labeled probe that paints all chromosomes except Chromosome 3 (Cambio, Cambridge, United Kingdom) were corrected for low level background by addition of human *Cot1* DNA (Roche; 1.7 or 3.3 μg/μl final concentration), denatured at 70 °C for 10 min, and reannealed at 37 °C for 30 min before hybridization. To obtain a probe that maps to the MHC class II locus, BAC RP11-399L10 (BACPAC Resources Centre) was purified after cesium chloride gradient centrifugation and labeled with biotin or rhodamine using a nick translation kit (Roche); unincorporated nucleotides were cleared using micro biospin P-30 chromatography columns (BioRad, Hertfordshire, United Kingdom). BAC probe was coprecipitated with human *Cot1* DNA before the addition of the chromosome paint and denaturation as above. Probe specificity was confirmed on human lymphocyte metaphase spreads.

After washing with PBS, cryosections were incubated at 37 °C with 250 μg/ml RNase A (1 h), treated with 0.1 M HCl (10 min), dehydrated in ethanol (30% to 100% series, 3 min each), denatured (8 min, 80 °C) in 70% deionized formamide in 2× SSC, and dehydrated as above, before probe was added. Hybridization was carried out at 37 °C for longer than 40 h. Posthybridization washes were as follows: 50% formamide in 2× SSC 42 °C (3× over 25 min), 0.1× SSC (60 °C, 3× over 30 min), and 4× SSC with 0.1% Tween-20 (42 °C, 10 min). Nuclei were counterstained with 2 μM TOTO-3 (Molecular Probes, Eugene, Oregon, United States) or 20 ng/ml DAPI (Sigma) in PBS/0.1% Tween-20. Coverslips were mounted in Vectashield (Vector Laboratories, Peterborough, United Kingdom) and EM grids were mounted in PBS and overlaid with a glass coverslip. As the hybridization signals were generally weaker in MRC5 cells, an additional incubation with 0.1% Triton X-100/0.1% saponin (10 min) was included before the HCl step, and denaturation time was increased to 12 min for these cells (no differences in signal-to-noise ratios were detected for chromosome painting in human lymphocytes in these conditions).

Mock-FISH was performed using hybridization buffer (50% deionized formamide, 10% dextran sulfate, 2× SSC, 50 mM phosphate buffer [pH 7.0]) in the absence of DNA probe.

### Immunolabeling

For immunolabeling, cryosections were washed in PBS, incubated in 0.1% Triton X-100 (10 min), and labeled as described previously [ 35], except that PBS+ contained 0.1% casein. For EM detection of CTs, rhodamine-labeled probes were indirectly immunolabeled using a rabbit anti-rhodamine antibody (1/500, 2 h; Molecular Probes), followed by a goat anti-rabbit IgG antibody conjugated with 10 nm gold particles (1/50, overnight; British BioCell, Cardiff, United Kingdom). FITC-labeled chromosome paints were detected using a biotin conjugated mouse anti-FITC antibody (1/1,000, 2 h; Jackson ImmunoResearch Laboratories, West Grove, Pennsylvania, United States), followed by a biotin-conjugated donkey anti-mouse IgG antibody (1/100, 1 h; Jackson ImmunoResearch), and a goat anti-biotin antibody conjugated with 5-nm gold particles (1/30, overnight; British BioCell). Control experiments in the absence of one of the paints showed insignificant cross-reactivity between antibodies. Grids were washed in PBS+ (4× over >3 h), rinsed in PBS, fixed in 0.5% glutaraldehyde in PBS (10 min), washed in distilled water, and incubated in 2% methylcellulose (10 min). Excess liquid was blotted and grids were left to dry.

For the biotin-labeled paint, FITC-conjugated streptavidin (1/500; Sigma) or AlexaFluor350 conjugated Neutravidin (1/100; Molecular Probes) were used. The biotin-labeled BAC probe for the MHC II locus was detected using rhodamine-conjugated neutravidin (1/500; Molecular Probes), followed by a biotin-conjugated goat anti-avidin antibody (1/500; Vector) and rhodamine-conjugated neutravidin. PML was detected with anti-PML rabbit IgG clone H238 (1/10; Santa Cruz Biotechnology), followed by an AlexaFluor 488–conjugated goat anti-mouse antibody (1/1,000; Molecular Probes). Histone H2B was detected with a rabbit anti-histone H2B polyclonal antibody (1/100; Chemicon), followed by a goat anti-rabbit antibody conjugated with 5-nm gold particles (1/50; British BioCell). Serine2-phosphorylated PolII was indirectly immunolabeled with H5 (1/1,000; Covance, Berkeley, California, United States). After immunolabeling and washing (3×) in PBS, antibodies were fixed (1 h) with 8% paraformaldehyde in 250 mM HEPES (pH 7.6), before mock-ISH or chromosome painting.

### Microscopy

For confocal laser scanning microscopy, images were collected sequentially on a Leica TCS SP2 (×100 PL APO 1.40 oil objective) equipped with argon (488 nm) and HeNe (543 nm; 633 nm) lasers or a Leica TCS SP1 (×100 PL APO 1.35 oil objective) equipped with UV (351/364 nm), argon (488 nm), krypton (568 nm), and HeNe (633 nm) lasers. For wide-field LM, images were collected sequentially on a Delta-Vision Spectris system (Applied Precision, Issaquah, Washington, United States) equipped with an Olympus IX70 wide-field microscope (×100 UPlanFl 1.3 oil objective), a charge-coupled device camera, and the following filters: DAPI, FITC, RD-TR-PE, CY-5, CFP, YFP. No bleed-through was detected in these conditions. The use of ultrathin cryosections allows for the use of wide-field microscopy with no reduction in axial *(z)* resolution and only a small reduction in lateral resolution [ 46].

For EM, images were collected on a JEOL 1011 transmission electron microscope (JEOL UK, Welwyn Garden City, Herts, United Kingdom) equipped with a cooled slow-scan KeenView charge-coupled device camera (1,392 × 1,024 pixels; Soft Imaging System, Münster, Germany).

### Image analysis and measurements

For LM experiments, images (TIFF files) were automatically merged using a MatLab script (kindly provided by Tiago Branco, University College London, London, United Kingdom), saved as new TIFF files, and manually thresholded in Adobe Photoshop (Adobe Systems, Edinburgh, United Kingdom) to define masks for nuclei or CTs. Threshold values were chosen empirically so that the entire CT was selected but no widespread nuclear background was included. Independent drawing of masks by four different people on 10 images were compared to test the reliability of this empirical method. Variability in CT volume was found to be 15%, and in intermingling volumes was 30%, in the same order of magnitude as the variability obtained across independent experiments. The values of the areas of these masks and the intersection between the masks for both CTs were extracted using another MatLab script (Tiago Branco).

CT and intermingling volumes were calculated according to stereological methods [ 47] after collecting random images of sections irrespective of their area and whether they contained CT signals (i.e., sections analyzed represented the whole nucleus). CT or intermingling areas were averaged across all sections and divided by the average of the nuclear areas. This ratio (R) is equivalent to the ratio of the respective average volumes, as shown here:



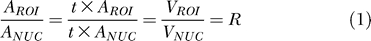



where *A _ROI_* is the average CT or intermingling area, *A _NUC_* is the average nuclear area, *V _ROI_* and *V _NUC_* are the corresponding average volumes, and *t* is the section thickness. Using average section volumes for *R* gives the same result as using average whole nuclei volumes if enough random sections from different cells are included in the calculation.

To obtain several values for *R* within one hybridization experiment (to allow statistical analysis), images were randomly grouped and *R* was calculated for each group. Standard deviations remained constant with increasing number of groups until a group size was reached at which *R* did not contain enough information and the standard deviation increased abruptly. The highest number of groups before this increase was used. Group size varied between different chromosome pairs, averaging 55 sections per group, and up to four groups were used in an experiment (a total of 57 to 211 sections were analyzed in individual experiments). Standard deviations obtained by this method were consistent with standard deviations between independent hybridization experiments. The *R* values were used for statistical tests, and considered to have a normal distribution, as normality plots for the analysis of residuals were positive. Two-sample comparisons were performed by two-tailed *t*-test and multisample comparisons by ANOVA. Regression analyses using an F-test were performed to test the significance of variable correlations. For the analysis of the MHC II locus data, we used Fisher's exact test for 2 × 2 contingency tables and chi-squared test for larger tables.

## Supporting Information

Figure S1 Fluorescence Intensity Profiles of the Images in Figure 1B– 1E
(469 KB JPG)

Figure S2 Additional Control Experiments Showing Preservation of Nuclear Structure during cryo-FISH (954 KB JPG)

Figure S3 Quantification of the Total Intermingling of Chromosome 3 with the Remaining Genome (214 KB JPG)

Figure S4 Graphical Analyses of the Distribution of Active RNA Polymerase II Sites within CTs and Areas of Intermingling (13 KB PDF)

Figure S5 Volumes for All CTs in Human Female Lymphocytes and Correlation with DNA Content (12 KB PDF)

Protocol S1 Quantification of Nuclear Volume That Contains Intermingled CTs (24 KB DOC)

Table S1 Interchange Yields in Phytohemagglutinin-Activated Human Lymphocytes (47 KB PDF)

## Abbreviations

3D: three-dimensional
CT: chromosome territory
DSB: double-strand break
EM: electron microscopy
FISH: fluorescence in situ hybridization
ICD: interchromatin domain
IFN-γ: interferon-gamma
LM: light microscopy
Pol: RNA polymerase
