# Supplementary material for: Intermingling of Chromosome Territories in Interphase Suggests Role in Translocations and Transcription-Dependent Associations
Source: PLoS Biol. 2006 Apr 25;4(5):e138. doi: 10.1371/journal.pbio.0040138 (PMC1440941; doi:10.1371/journal.pbio.0040138)
Supplement: Supplementary file 1 [file pbio.0040138.sg004.pdf]

Branco, Figure S4

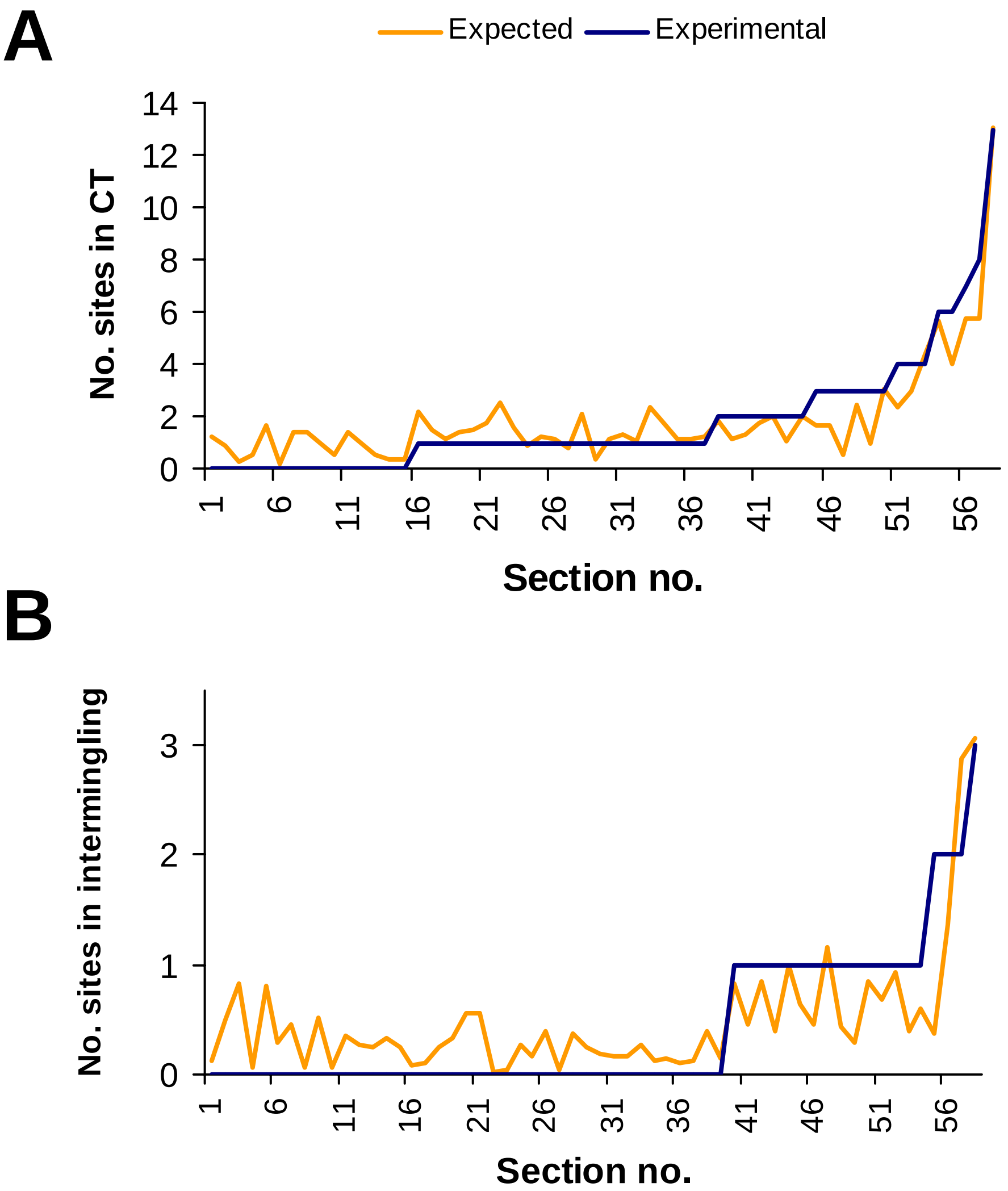

Figure S4. Graphical analyses of the distribution of active RNA polymerase II sites within CTs and areas of intermingling. The number of serine 2-phosphorylated Pol II sites (as shown in Fig. 3) colocalizing with chromosome 3 territory (**A**) or with areas of intermingling between chromosome 3 and all other chromosomes (**B**) were plotted for each section (blue line). The average site density of each nuclear section was used to predict the number of sites in each of these areas, as expected from a uniform distribution (yellow line). Good correlation is observed with the experimental values (R=0.913 for chromosome 3 and R=0.776 for the intermingling area), showing that there is no enrichment or depletion of active Pol II sites in the CT or intermingling areas.
