# Supplementary material for: Intermingling of Chromosome Territories in Interphase Suggests Role in Translocations and Transcription-Dependent Associations
Source: PLoS Biol. 2006 Apr 25;4(5):e138. doi: 10.1371/journal.pbio.0040138 (PMC1440941; doi:10.1371/journal.pbio.0040138)
Supplement: Supplementary file 2 [file pbio.0040138.sg005.pdf]

Branco, Figure S5

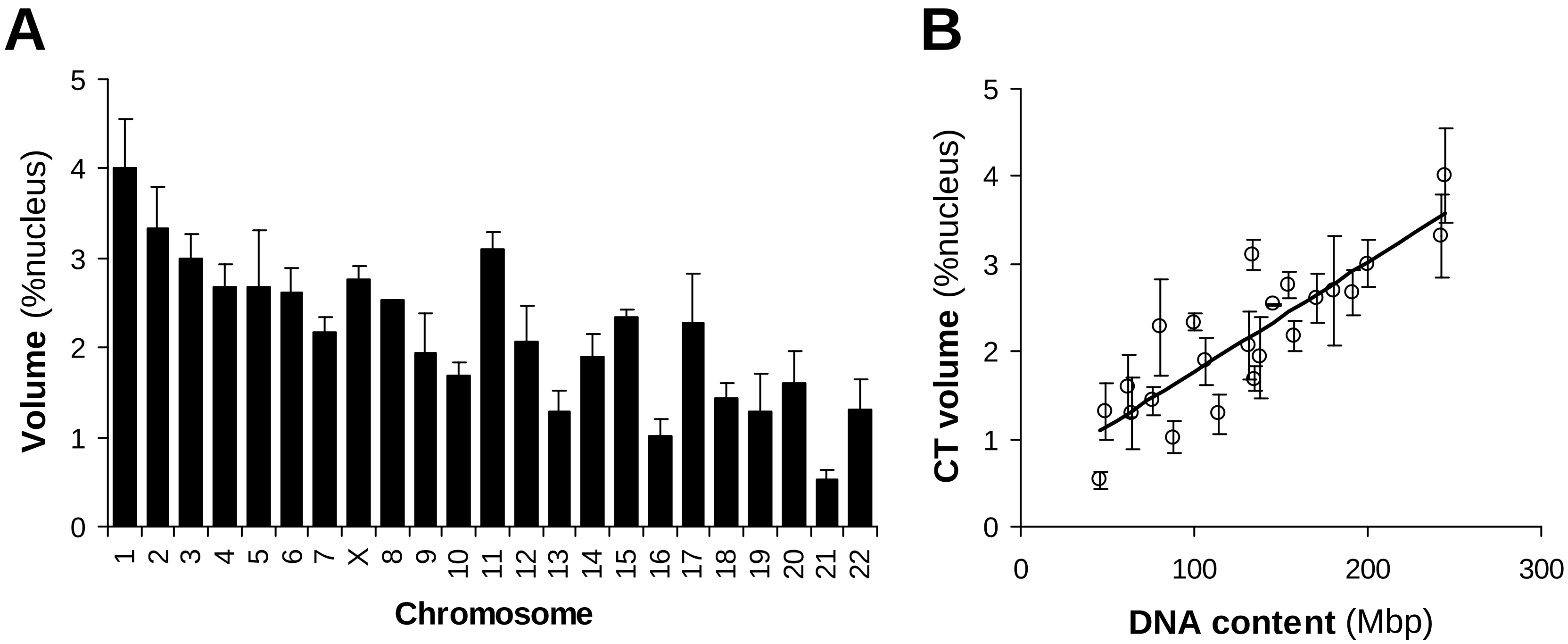

Figure S5. Volumes for all human female CTs in lymphocytes and correlation with DNA content. CT volumes were measured (**A**) and plotted against their respective DNA content (**B**). Despite the good correlation, there are significant deviations from the regression line in several data points, which results in distinct DNA compaction ratios for different CTs.
