# Supplementary material for: Intermingling of Chromosome Territories in Interphase Suggests Role in Translocations and Transcription-Dependent Associations
Source: PLoS Biol. 2006 Apr 25;4(5):e138. doi: 10.1371/journal.pbio.0040138 (PMC1440941; doi:10.1371/journal.pbio.0040138)
Supplement: Supplementary file 3 [file pbio.0040138.st001.pdf]

Branco, Table S1

Table S1. Interchange yields in phytohemagglutinin-activated human lymphocytes. Arsuaga and colleagues [26] measured interchange yields in activated human lymphocytes after treatment with ionizing radiation and M-FISH analysis. Original values were recalculated here as a percentage of the total number of metaphases analyzed (n=3585). Intermingling volumes were measured (in the same cell type) for pairs of chromosomes that span the range of frequencies (0.08 – 1.23%; highlighted in red).

Chromosome	**2**	**3**	**4**	**5**	**6**	**7**	**8**	**9**	**10**	**11**	**12**	**13**	**14**	**15**	**16**	**17**	**18**	**19**	**20**	**21**	**22**
**1**	1.23	1.06	1.17	0.81	0.73	0.81	0.50	1.09	0.81	0.70	0.50	0.42	0.50	0.95	0.86	0.61	0.33	0.39	0.61	0.25	0.75
**2**		1.20	1.03	0.89	0.84	0.67	0.70	0.81	0.45	0.67	0.84	0.81	0.25	0.73	0.22	0.67	0.22	0.20	0.33	0.36	0.42
**3**			0.59	0.86	0.89	0.67	0.59	0.73	0.64	0.70	0.64	0.59	0.50	0.50	0.53	0.59	0.31	0.47	0.31	0.33	0.28
**4**				0.64	0.75	0.58	0.67	0.73	0.56	0.36	0.53	0.64	0.61	0.56	0.45	0.50	0.31	017	0.33	0.28	0.20
**5**					0.47	0.86	0.73	0.70	0.67	0.84	0.70	0.70	0.42	0.53	0.22	0.53	0.36	0.20	0.45	0.20	0.11
**6**						0.50	0.61	0.59	0.86	0.36	0.84	0.50	0.42	0.53	0.39	0.42	0.36	0.28	0.25	0.22	0.20
**7**							0.56	0.56	0.47	0.78	0.70	0.36	0.50	0.22	0.50	0.64	0.31	0.25	0.53	0.17	0.20
**8**								0.36	0.33	0.67	0.31	0.70	0.42	0.45	0.33	0.45	0.47	0.11	0.25	0.20	0.22
**9**									0.59	0.70	0.20	0.64	0.64	0.75	0.56	0.42	0.61	0.22	0.25	0.20	0.28
**10**										0.50	0.59	0.39	0.39	0.28	0.53	0.39	0.25	0.14	0.31	0.20	0.08
**11**											0.70	0.14	0.42	0.45	0.53	0.42	0.22	0.28	0.33	0.08	0.31
**12**												0.25	0.45	0.25	0.33	0.45	0.22	0.36	0.28	0.14	0.14
**13**													0.81	0.28	0.28	0.20	0.45	0.14	0.17	0.20	0.25
**14**														0.61	0.36	0.17	0.28	0.06	0.17	0.36	0.31
**15**															0.61	0.36	0.25	0.20	0.31	0.20	0.25
**16**																0.33	0.42	0.33	0.56	0.22	0.36
**17**																	0.14	0.11	0.31	0.14	0.28
**18**																		0.06	0.31	0.25	0.08
**19**																			0.17	0.00	0.28
**20**																				0.20	0.28
**21**																					0.17
