# Supplementary material for: Intermingling of Chromosome Territories in Interphase Suggests Role in Translocations and Transcription-Dependent Associations
Source: PLoS Biol. 2006 Apr 25;4(5):e138. doi: 10.1371/journal.pbio.0040138 (PMC1440941; doi:10.1371/journal.pbio.0040138)
Supplement: Supplementary file 4 [file pbio.0040138.sd001.doc]

**Branco, Protocol S1**

## Quantification of nuclear volume that contains intermingled CTs.

To estimate the total extent of CT intermingling in the nucleus we made use of the correlation observed between chromosome translocations and intermingling volumes, and used two independent calculations using either the intermingling volumes measured between pairs of chromosomes or the intermingling volume between chromosome 3 and all others.

In the first approach, the intermingling volume for each autosome pair was extrapolated from the regression curve (Fig. 2B) using the translocation frequencies in Table S1 (these data do not include translocations involving the sex chromosomes). After summing the extrapolated values we find that 17% of the nuclear volume contains intermingled sequences from different CTs. To include the contribution of two X chromosomes in female lymphocytes we calculated an average intermingling volume and extrapolated for the whole genome, as follows:

1. For our sample of chromosome pairs analyzed, the average intermingling volume per pair was calculated (0.091% of the nuclear volume);
2. To include the X chromosomes this value was multiplied by all of the pair-wise combinations between 23 homologs (i.e. 253), resulting in a value of total intermingling of 23% of the nuclear volume;
3. As our sample may under or over-represent the average for the whole genome, we next estimated a “correction factor” by assuming that our sample has a similar bias in terms of translocation frequencies. Therefore, we used the data on translocation frequencies (Table S1) to calculate the average translocation frequency for our sample of autosome pairs (0.54%), and multiplied by the 231 combinations of 22 autosomes, resulting in a frequency of 125.6%. As the real total frequency of translocation is 104.2% (calculated from Table S1), our sample of autosome pairs overestimates the total frequency by a factor of 1.2;
4. We can now use this factor to estimate the true intermingling volume in the whole nucleus, if we assume that the inclusion of data on translocations involving the X chromosome on the latter step would not significantly affect the “correction factor”. Dividing 23% (obtained in step 2) by the “correction factor” 1.2 gives 19%.

This value does not take into account inter-homolog and multiple chromosome intermingling. The former increases the total intermingling volume, whereas the latter decreases it (we measured a triple intermingling volume of only 0.01% of the nucleus between chromosomes 1, 2 and 3).

In the second approach, similar calculations were done using the intermingling volume of chromosome 3 with all others (2.41% of the nuclear volume). Since all CTs potentially intermingle with chromosome 3, an average intermingling volume of chromosome 3 with any other CT was calculated (0.105% of the nuclear volume), and steps 2 to 4 were followed. The uncorrected value was 26.5%, which after dividing by the respective “correction factor” (1.4) also results in a value of 19% for the total intermingling in the nucleus.
